# *Drosophila melanogaster* resilin improves the mechanical properties of transgenic silk

**DOI:** 10.1371/journal.pone.0282533

**Published:** 2023-03-03

**Authors:** Shuo Zhao, Xiaogang Ye, Xiangping Dai, Xinqiu Wang, Shihua Yu, Boxiong Zhong

**Affiliations:** 1 Department of Laboratory Medicine, The First Affiliated Hospital of Henan University of Chinese Medicine, Zheng Zhou, Henan, People’s Republic of China; 2 College of Animal Sciences, Zhejiang University, Hangzhou, Zhejiang, People’s Republic of China; East China Normal University School of Life Sciences, CHINA

## Abstract

Resilin is a natural protein with high extensibility and resilience that plays a key role in the biological processes of insects, such as flight, bouncing, and vocalization. This study used *piggy*Bac-mediated transgenic technology to stably insert the *Drosophila melanogaster* resilin gene into the silkworm genome to investigate whether exogenous protein structures improve the mechanical properties of silkworm silk. Molecular detection showed that recombinant resilin was expressed and secreted into silk. Secondary structure and mechanical property analysis showed that the β-sheet content in silk from transgenic silkworms was higher than in wild-type silk. The fracture strength of silk fused with resilin protein was 7.2% higher than wild-type silk. The resilience of recombinant silk after one-time stretching and cyclic stretching was 20.5% and 18.7% higher than wild-type silk, respectively. In summary, *Drosophila* resilin can enhance the mechanical properties of silk, and this study is the first to improve the mechanical properties of silk using proteins other than spider silk, which broadens the possibilities for the design and application of biomimetic silk materials.

## Introduction

Resilin is an elastomer found in the cuticle of most insects and was first described in locusts by Weis Fogh in the early 1960s [1]. It consists of randomly oriented coiled polypeptide chains with high flexibility and fluidity. The polypeptide chains are connected by covalent crosslinking via di-tyrosine and tri-tyrosine [2]. Crosslinked resilin is very stable and has physical characteristics similar to rubber, such as low stiffness, high ductility, high energy storage, and excellent elasticity and fatigue life. These characteristics play crucial roles in insect biological processes, such as flight, bounce, and sound [3–6]. Resilin’s performance has attracted the attention of researchers. In 2005, Elvin et al. expressed the *Drosophila* resilin gene in *Escherichia coli* for the first time, and the recombinant protein exposed to photochemical crosslinking exhibited rebound performance exceeding that of artificial rubber [7]. Subsequently, researchers designed and constructed multiple recombinant proteins based on the resilin sequence, tailoring the protein to exhibit additional biological characteristics. Resilin derivatives have been incorporated into numerous applications, including drug delivery, tissue engineering, and biosensors. [8–13].

Silkworm (*Bombyx mori*) silk has been used in the textile industry for thousands of years, and it is the only natural silk fiber that can be produced on a large scale [14]. Silk is composed of two independent silk fibers. Each fiber contains a silk fibroin core wrapped by hydrophilic sericin. Silk fibroin is a complex consisting of silk fibroin heavy chain (FH), silk fibroin light chain (FL), and P25 that are connected via covalent or noncovalent bonds [15, 16]. FH is a hydrophobic, high-molecular-weight protein with many (GA) NGX repeat motifs, which can form β-sheet crystals and may be the molecular basis of the mechanical properties of silk [17, 18].

Silkworm silk is light, breathable, nontoxic, easy to dye, and an ideal textile material [19]. However, its low mechanical strength limits its application outside the textile field. In recent years, attempts to enhance the physical properties of silk through transgenic technology have made some progress. For example, Tuele et al. first integrated the recombinant spider silk gene into the silkworm genome using a *piggy*Bac transposon and obtained silk with improved mechanical properties [20, 21]. Kuwana et al. used the FH promoter to drive the expression of a recombinant spider silk gene in silkworm silk glands, and the maximum stress of the obtained silk/spider silk fusion fiber reached 600 MPa [22]. You et al. introduced recombinant spider silk protein genes of different lengths into the silkworm genome and increased the silk’s strength by 72% relative to the control silk [17]. Zhao et al. expressed recombinant spider silk protein containing different numbers of polyalanine motifs in the silk gland of silkworm. The polyalanine structure improved the strength and modulus of the resulting silk fibers [23]. Tang et al. fused spider pyriform silk and aggregate silk genes with the silk light chain gene. The toughness of the transgenic silk fiber increased by 91.5%, and the maximum stress increased by 36.9% [24]. These studies show that introducing foreign proteins into silk and optimizing the molecular structure by gene editing technology is an effective method to improve the mechanical properties of silk fibers. The fusion of a spider silk protein and silk fibroin enables the resulting protein to participate in the silk fibroin complex assembly and secondary structure formation, significantly affecting the silk’s mechanical properties [23]. However, to our best knowledge, no studies that improve the mechanical properties of silk with proteins other than spider silk have been published to date.

In this study, we designed a fusion gene from *Drosophila* resilin and FL to investigate which molecular structures improve the mechanical properties of silk. The recombinant gene was integrated into the silkworm genome by *piggy*Bac transposition technology, and the performance and structure of the transgenic silk were analyzed. Our results show that incorporating recombinant resilin into transgenic silk contributes to forming β-sheet structure and improves the strength and elasticity of transgenic silk.

## Materials and methods

### Animals

The multivoltine non-diapause silkworm strain Lan10 used in the experiment was preserved by the Silkworm Genetics Laboratory of Zhejiang University. Silkworms were fed with fresh mulberry leaves at 25°C and 80% relative humidity.

### Construction of *piggy*Bac transposon vector

The resilin gene *CG15920* of *D*. *melanogaster* (with 3 exons in the full length) was synthesized by GeneScript (Nanjing, China). *Piggy*Bac transposon plasmid pBac-dsRed-FL [IE1-dsRed-SV40-FL promoter-FL exons (1–7)-Xmal-NotI] was preserved in our laboratory. The pBac-dsRed-FL were double digested to recover fragment pBac-dsRed-FL [IE1-dsRed-SV40-FL promoter-FL exons (1–7)-Xmal-NotI]. Then, the *CG15920* sequence was ligated to the fragment and obtain the *piggy*Bac vector pBac-resilin containing the fusion gene of FL exons 1–7 and the resilin [IE1-dsRed-SV40-FL promoter-FL exons (1–7)- *CG15920*–FL-poly (A)].

### Silkworm transformation and identification

The wild-type (WT) silkworm Lan10 insects were used for transgenic experiments according to a previously described method [25]. The preparation of the *piggy*Bac transposition system was also performed according to our previously described method [17]. Briefly, the *piggy*Bac plasmid and helper plasmid were mixed at a ratio of 1:1.5 and injected into fertilized Lan10 eggs within 2 h after oviposition. After the microinjection experiment, the embryos were incubated at 25°C for 7–10 days. The larvae were reared with fresh mulberry leaves at 25°C and 80% humidity. The adults were hybridized with the WT, and the larvae of the G1 generation were observed with a fluorescence microscope (Olympus, Japan) to screen the positive silkworm individual. The positive G1 larvae were fed fresh mulberry leaves at 25°C and 80% humidity and then hybridized with the wild-type at the adult stage. At the fifth instar of G2, the positive silkworm lines with the brightest fluorescence were selected for this study.

### Insertion site analysis

The *piggy*Bac transposon randomly inserts the sequences between transposable arms into the silkworm genome under the action of transposase (helper plasmid). Primers were designed according to the two transposable arm sequences and amplified by reverse PCR [23]. The amplified products were connected to the pMD-19T vector and sequenced. The sequencing results were compared with the sequences in the silkworm genome database kaikobase (http://sgp.dna.affrc.go.jp/index.html) to determine the position of the insertion site on the silkworm chromosome.

### Western blot and silver staining analysis

Soluble silk fibroin from the transgenic cocoon shells was prepared as previously described [23]. A sample containing 40 μl of the total protein was loaded and separated by 4–15% gradient SDS–PAGE. After separation, the protein was blocked onto PVDF membrane using a GE transfer cell according to the manufacturer’s instructions. The PVDF membrane containing the target protein was blocked with 5% nonfat milk powder for 2 hours. Then, the specific polyclonal antibody of resilin (diluted at 1:8000) was used as the primary antibody (GenScript, Nanjing, China), and peroxidase-conjugated goat anti-rabbit IgG-HRP (1:8000 dilution) was used as the secondary antibody (GenScript, Nanjing, China). The primary antibody was specific to the “CGAPGQNQKPSDSYG” sequence corresponding to exon 1 of the *CG15920* gene. ECL luminescence reagent (Sangon Biotech, Shanghai, China) was used for signal detection. Silver staining analysis was performed according to the instructions of a silver staining kit (Beyotime, Shanghai).

### FTIR spectroscopy analysis

The cocoons were cut into pieces with a diameter of 1 mm and soaked in 1 L Na_2_CO_3_ (0.5 wt.%) for every 10 g of cocoon pieces for 30 min at 95°C to remove the sericin on the silk surface. Then, the obtained silk fibroin fiber was washed with deionized water and dried at room temperature. A total of 2 mg of dried degumming silk was mixed with 200 mg of potassium bromide (KBr), ground thoroughly in a mortar, and then dried. The mixture was molded into tablets for testing. The Fourier Transform Infrared (FTIR) spectra were recorded using a FTIR (FTIR-8400S, Shimadzu, Tokyo, Japan). Omnic software was used for baseline correction and data smoothing. Origin software was used for peak separation and peak combination to calculate the semiquantitative data of the secondary structure of silk fibroin. Gaussian function was used for multi-peak fitting and the error in the fit parameters was assessed by the adjusted R squared.

### Mechanical properties testing

Immerse the cocoon in deionized water at 80°C for more than 12 hours. When the cocoon was loose, the single silk was cut off for subsequent performance tests. Five cocoons were selected from each variety randomly, and five monofilaments were taken from each cocoon for measurement. Each single silk fiber was glued (glue502) across a rectangular frame, which was cut into a cardboard support to obtain a gauge length of 30mm. The initial length (L0) of each silk fiber was 30mm. The cross-sectional diameter of each silk sample was measured with a digital microscope (VHX-600, Keyence, Osaka, Japan) at 1000× magnification. Each sample was measured 3 times, and the average value was taken to calculate the cross-sectional area of each sample. Then, the mechanical properties of silk samples were calculated with the cross-sectional area as a parameter. The AGS-J Universal Test instrument (Shimadzu, Tokyo, Japan) was used, and a 5 N load cell was equipped for tensile testing with a constant rate of 2 mm/min.

Then, the elastic properties of silk were tested. According to the stress-strain test results, three strain groups are set, which were 5%, 10% and 15%. The stretching speed is 5 mm/min, and the initial distance is 30 mm. The fatigue test is set with 60 stretching times, and a 5 N sensor was used. The stretching speed is 5 mm/min, the initial distance is 30 mm, the upper vertex and the lower vertex stay for 1 s, and the stretching distance is set to 5%, 10% and 15%.

The calculation formula of elastic recovery rate is:

$$R=\frac{L-L\prime }{L}\times 100\%$$ (1)

where R is the elastic recovery rate, L is the total elongation and L’ is the residual elongation.

### Statistical analyses

GraphPad Prism 7 and SPSS 23 were used for statistical analysis. The error bar in this study represents the standard deviation of the repeat group. Two-tailed unpaired t-tests were used to compare data sets.

## Results

### Construction of transgenic plasmids and screening of transgenic silkworms

**Fig 1:**
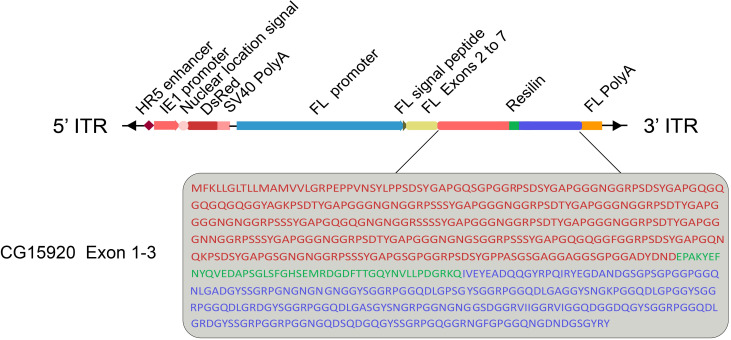
Schematic representation of resilin transgenic vector. 5’ITR and 3’ITR are the sequence of the left and right arms of the *piggy*Bac transposon plasmid. The IE1 promoter activates the systemic expression of DsRed as a marker for screening positive silkworms.

*CG15920* was the first resilin gene identified [7]. In this study, a complete *CG15920* gene was designed to maintain the mechanical properties of the natural resilin protein as much as possible. *CG15920* is 1785 bp long and encodes 595 amino acids. Codon preferences in the silk fibroin proteins FL and FH were analyzed, and the results were used to optimize codon usage in designing *CG15920*. We then fused resilin with the silk FL chain and expressed the fusion gene using an FL promoter to exploit the self-assembly behavior of silk fibroin to incorporate foreign proteins into silk molecules. A red fluorescent protein gene (DsRed) with an IE1 promoter was used as a screening marker (Fig 1). Transgenic silkworms were selected from the G1 generation by screening for systemic red fluorescence, and silkworm cocoons were used to detect silk protein and characterize the mechanical performance.

### Insertion site analysis

**Fig 2:**
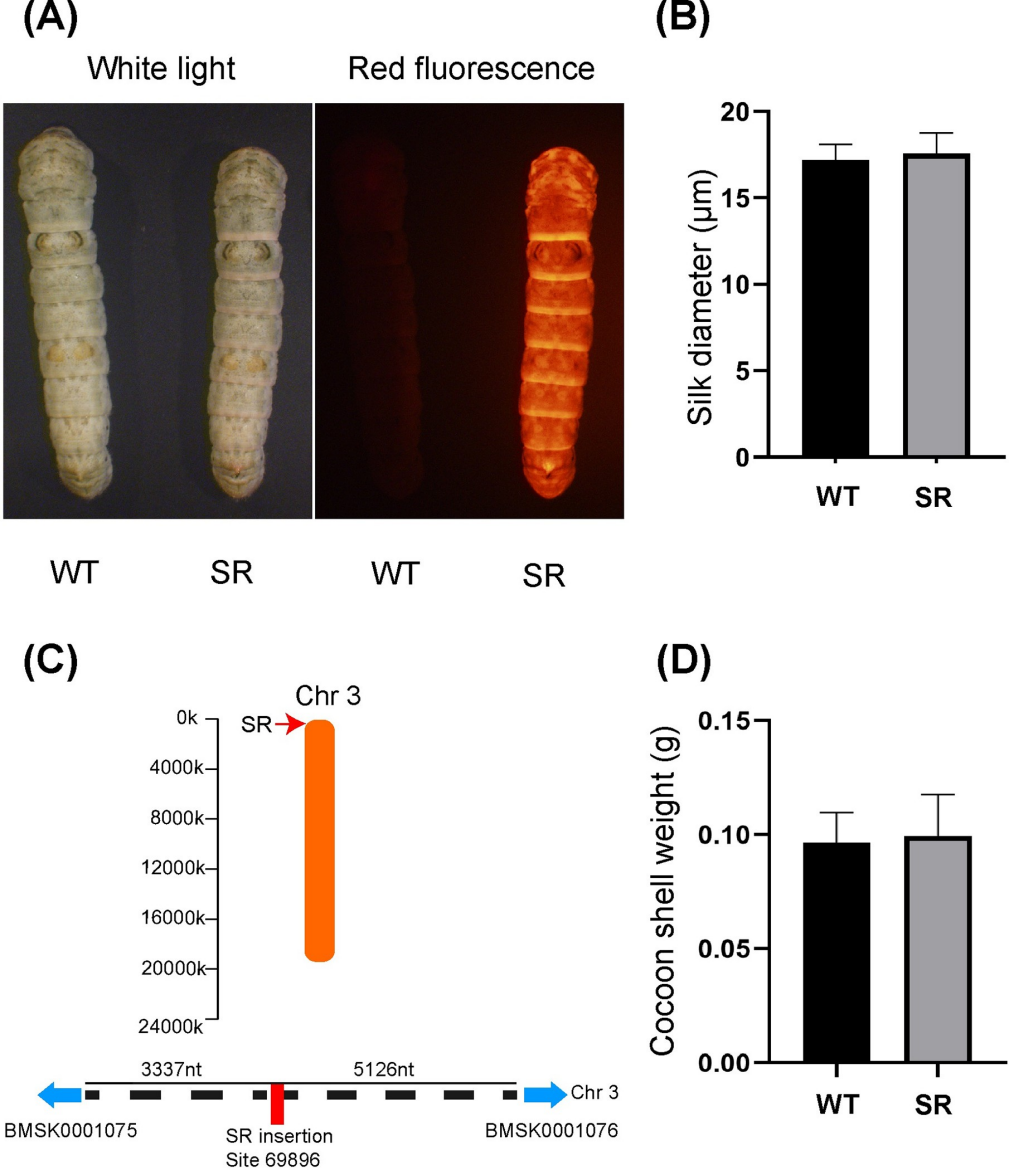
Screening and identification of transgenic positive silkworm. (A) The fluorescent phenotype of the DsRed expressed on the whole body of silkworm. (B) Statistics of silk diameter. (C) Locations of the insertion site of the transgenic silkworm on the chromosome. (D) Statistics of cocoon weight.

The selected positive silkworm line (SR) was hybridized with the wild type (WT) to obtain the G3 generation (Fig 2A). Silkworms that did not express fluorescent protein were eliminated, and the remaining positive silkworms were used as experimental materials in this study. *Piggy*Bac transposons randomly insert into TTAA sequences of the silkworm genome. DNA was extracted from 3 SR silkworm heads, and insertion site analysis was performed using reverse PCR (Fig 2C). SR silkworms contain a single transposon insertion in a noncoding region of the silkworm genome. No differences in growth or development were observed between SR and WT silkworms during feeding, and SR silkworms exhibited normal silk synthesis and secretion (Fig 2D). Digital microscope analysis shows no difference in the diameter of silk between SR and WT silks (Fig 2B).

### Identification of resilin expression in transgenic silkworms

**Fig 3:**
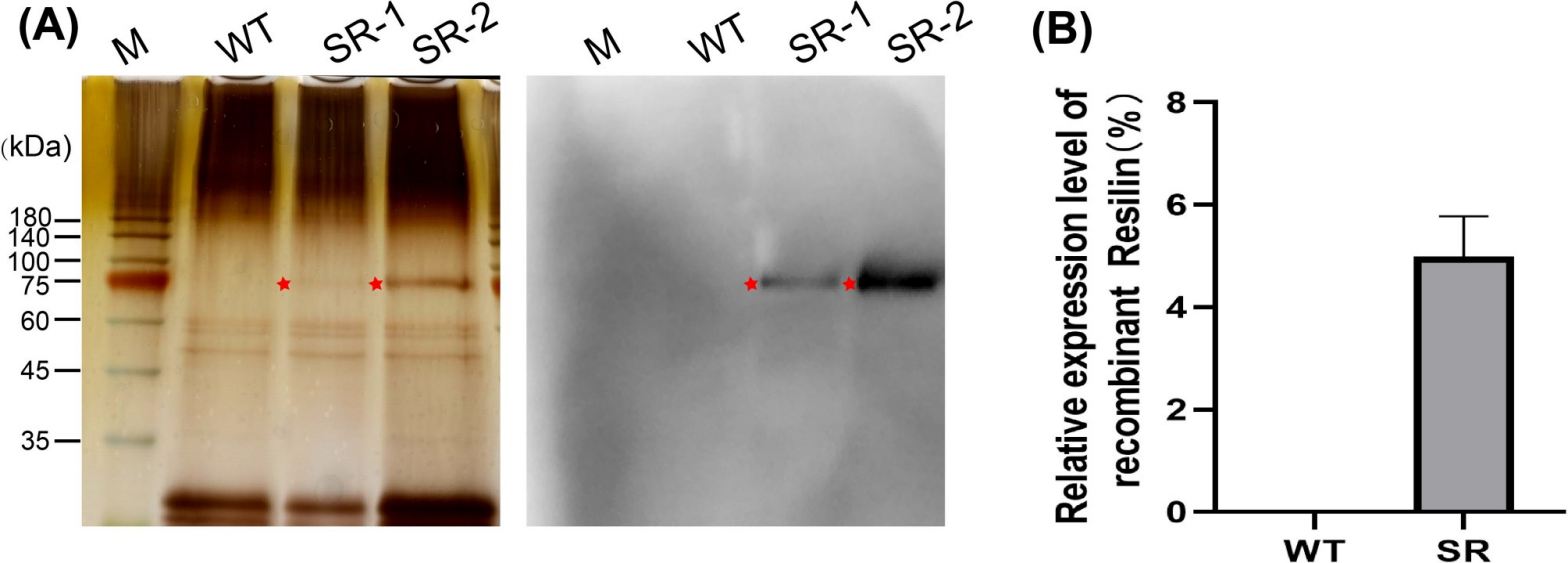
Expression identification and analysis of the recombinant resilin in the silkworm cocoon shells. (A) The recombinant resilin in cocoon shell were detected by silver staining (left) and Western-blot (right). WT: the wild-type silkworm Lan10. SR-1 and SR-2: different individuals of the transgene-positive silkworm germ line. (B) Expression analysis of the foreign protein relative to the FL protein through gray analysis using the Gel-Pro-analyzer4 software. WT: the wild-type silkworm Lan10. SR: the transgene-positive silkworm germ line. The target protein band is marked by an asterisk.

The theoretical molecular weight of the foreign protein carried by SR is 83.37 kDa. Silk fibroin was extracted by dissolving cocoon shells with lithium bromide and isolating proteins by dialysis, followed by SDS-PAGE electrophoresis. Silver staining shows a protein band corresponding to the size of the target protein above the 75 kDa marker (Fig 3A). Immunoassays with antibodies specific to the first exon of *CG15920* detected a band at the same position as silver staining (Fig 3A), verifying the expression of the foreign protein. The proportion of resilin fusion protein relative to silk fibroin light chain using protein band gray analysis is 4.98%.

### Secondary structure of transgenic silk

**Fig 4:**
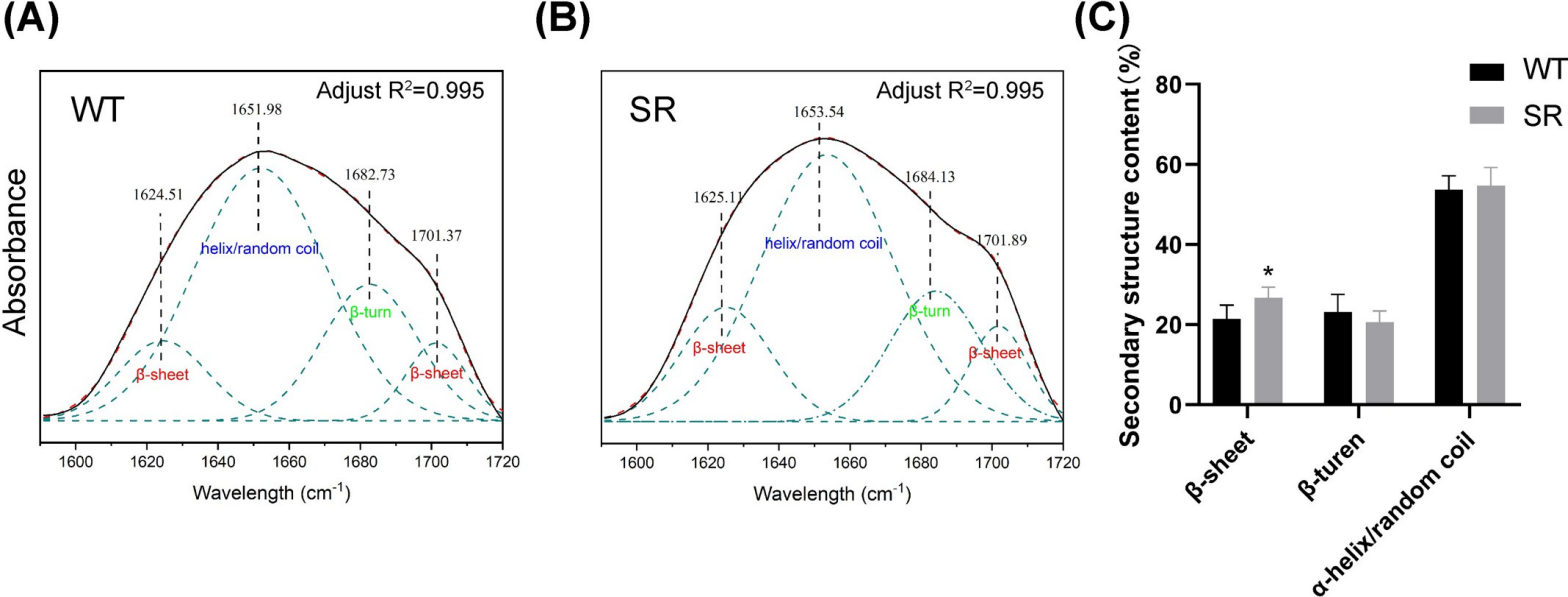
Characterization of secondary structure of transgenic silk. (A, B) Deconvolution of the corresponding amide I band of the WT. (C) Secondary structure content of the control and SR. The mean ± SD derived from three independent replicate experiments. * represent p < 0.05, (Two-tailed unpaired t-tests).

Fourier transform infrared spectroscopy (FTIR) is typically used to analyze the secondary structure of silk fibers. The amide I band of SR silk was analyzed by peak deconvolution. Deconvoluted peaks appearing at 1615–1645 cm^-1^ and 1690–1700 cm^-1^ represent β-sheet structures; peaks at 1645–1665 cm^-1^ represent α-helical/disordered coiled structures, and peaks at 1680–1690 cm^-1^ represent β-turn structures [26–28]. After deconvolution, the secondary structure distribution in transgenic silk was obtained by quantitative analysis of the subpeak area. The results are the average of three independent tests and showed that the contents of β-sheets, β-turns, and α-helical/disordered coils in the SR silk are 26.71%, 20.05%, and 54.73%, respectively (Fig 4). The contents of β-sheets in the WT silk are 21.4%. Resilin increases the β-sheet content in SR silk, whereas the β-turn and α helix/random coil contents in SR and WT silks were similar.

### Mechanical properties of transgenic fibers

**Fig 5:**
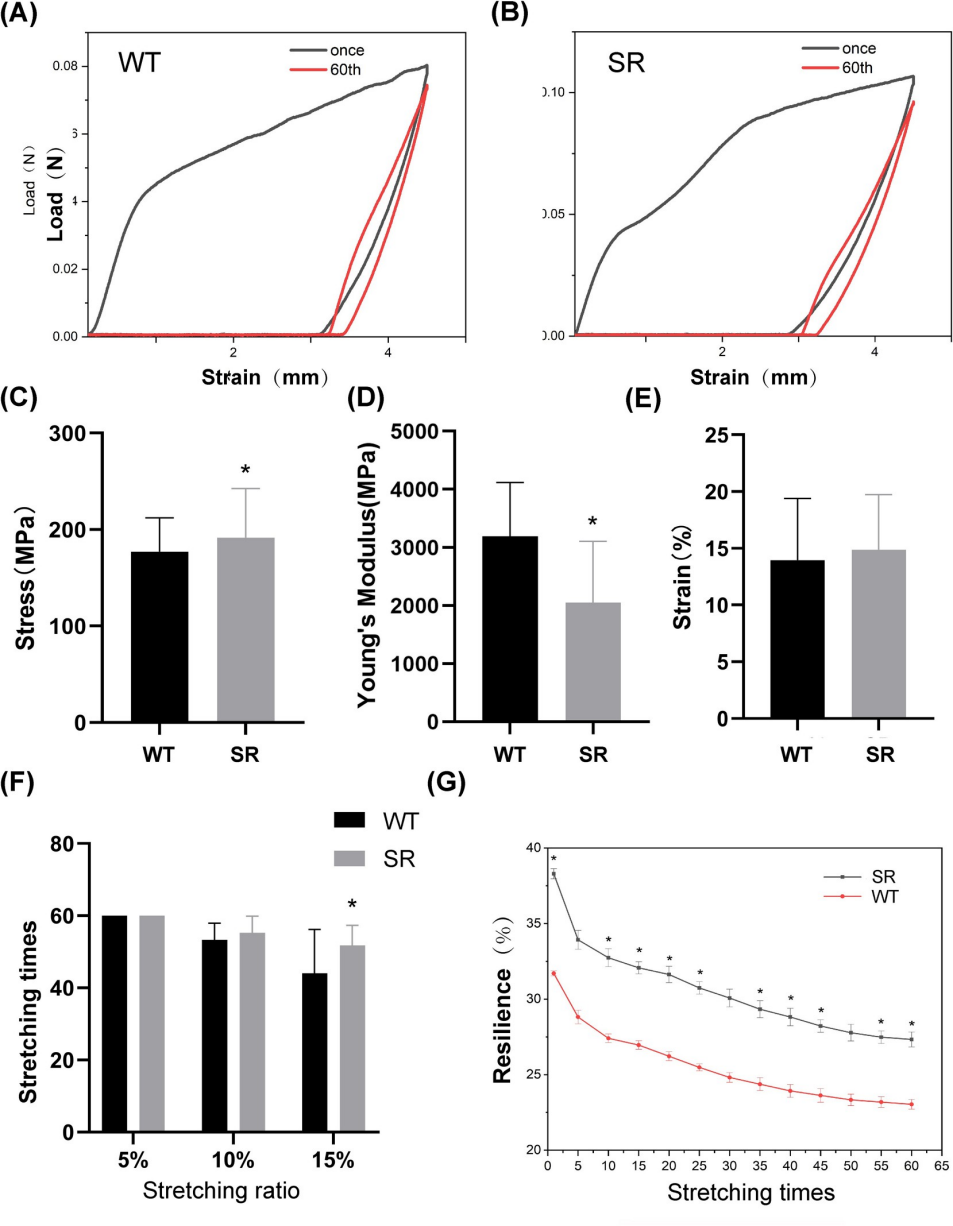
Mechanical properties of the recombined resilin silk fibers from the SR strains. (A, B) Stress–strain curves of WT and SR silk. (C-E) Graphs of the maximum stress, toughness and Young’s modulus, respectively. (F) Stretching times of SR and WT at different stretching ratios. (G) 15% cyclic tensile test for SR and WT. The mean ± SD derived from three independent replicate experiments. * represent p < 0.05, (Two-tailed unpaired t-tests).

Mechanical properties of SR and WT silks were measured using 25 monofilaments selected from 5 SR or WT cocoons (Fig 5A and 5B). We harvested SR and WT silks during identical feeding seasons to ensure accurate comparisons and avoid any influence from environmental variability on silk strength. Tests were conducted under identical conditions, and the data were showed in S1 File. The presence of resilin had a significant impact on the strength and Young’s modulus of transgenic silk. The stress of SR silk (190.378 MPa) was 7.2% higher than that of WT silk, and its Young’s modulus (2088.48 MPa) was 36.4% lower than that of WT silk (Fig 5C–5E).

Cyclic tensile tests were conducted on SR and WT silks at 5%, 10%, and 15% tensile ratios (Fig 5F and 5G). SR and WT silks experience similar tensile times at 5% and 10% tensile ratios, whereas at a 15% ratio, SR silk has higher breaking tensile times than WT silk. We then set the tensile length at 15% to test the recovery performance of silk in the ultimate tensile state and measured the average of 5 successful tests. The tensile recovery rate of SR is 38.2%, and that of WT is 31.7%. The recovery rate of SR silk is 20.5% higher than that of the control. Tensile recovery for each silk decreases gradually as the number of stretching cycles increases. At 60 cycles, the decreases in tensile recovery for SR and WT are 27.3% and 23%, respectively.

## Discussion

Natural evolution has produced numerous biological proteins with excellent performance, providing materials and inspiration for humans to develop high-performance materials [29]. Resilin is a well-known mechanically functional protein in arthropods, second only to spider silk [2]. This study describes the successful transfer of a fusion gene of Drosophila resilin and FL into the silkworm genome by *piggy*Bac-mediated transposition technology. A fluorescent marker gene was used to screen the G1 hybrid obtained by backcrossing with WT silkworms. The target protein was detected in the cocoon shell of G2 silkworms, indicating that the foreign protein was successfully expressed and secreted into the silk.

GAGAGS hexapeptide repeats in silk proteins form a microcrystalline region through an antiparallel β-sheet, which is the primary molecular basis of silk’s mechanical strength [30, 31]. Mechanical tests showed that the tensile strength of SR silk is 7.2% higher than that of WT silk, which is consistent with an increase in β-sheet content. Dragline silk has the best mechanical properties among spider silk types and comprises two silk fibroin proteins, MaSp1 and MaSp2 [32, 33]. Current thinking is that MaSp1 is rich in poly (A) or poly (GA) motifs that contribute to spider silk strength and modulus by forming β-sheet crystals via glycine-mediated hydrophobic interactions. MaSp2 is rich in a related proline motif, GPGXX [32–35]. Some scholars found that GPGXX interferes with the tight packing between crystals. This interference introduces water molecules and disrupts the order of glycine-rich motifs, leading to a decrease in the initial modulus of spider silk [36, 37]. Resilin is a proline-rich protein with 17 GGRPSDSYGAPGGN proline-rich motifs in the first exon and 12 GYSGGRPGGQDLG proline-rich motifs in the third exon. In this study, Young’s modulus of SR silk decreased by 36.4% compared with WT silk. We speculate that this may be due to a large amount of proline in the recombinant protein.

Tensile tests showed that SR silk exhibited 20.5% and 18.7% higher resilience than WT silk after one-time stretching and cyclic stretching, respectively, indicating that the mechanical properties of the transgenic silk tend to change as they do in the natural resilin. Previous studies typically focused on improving the mechanical properties of silk by integrating the spider silk protein, which has excellent mechanical strength and molecular structure characteristics like silk [20–24]. For example, spider silk and silk’s primary structures contain multiple repeat sequences, high GC content, similar codon preferences, and similar glycine and alanine contents. In addition, silkworms and spiders have similar silk secretion processes that may be critical to forming secondary structures and conferring mechanical properties in silk proteins [38–41]. These molecular structures and spinning behavior similarities make spider silk an ideal foreign protein for improving and strengthening silk. However, other protein molecules have excellent performance or unique functions in nature. It is unknown whether these protein molecules can improve the mechanical properties of silk fibers after undergoing secondary structure reconstruction during silk fibroin molecule assembly and secretion. In this study, a resilin protein from insect cuticles was introduced into silk fibroin. We showed that the resilin incorporation improved the mechanical properties of transgenic silk and had no impact on the survival and silk secretion of transgenic silkworms relative to WT. This study represents a meaningful attempt to improve the mechanical properties of silk by using proteins other than spider silk.

In conclusion, the presence of resilin significantly improves silk’s strength and resilience and effectively improves silk’s mechanical properties. In this study, biological proteins other than spider silk were used to optimize the molecular structure of silk for the first time, and good results were obtained, providing new inspiration for the study of silk performance improvement.

## Supporting information

S1 File This file contains the raw data obtained for each mechanical test. (XLSX)

S1 Raw images (TIF)
