# Supplementary figures and images for: Drosophila melanogaster resilin improves the mechanical properties of transgenic silk
Source: PLoS One. 2023 Mar 3;18(3):e0282533. doi: 10.1371/journal.pone.0282533 (PMC9983856; doi:10.1371/journal.pone.0282533)

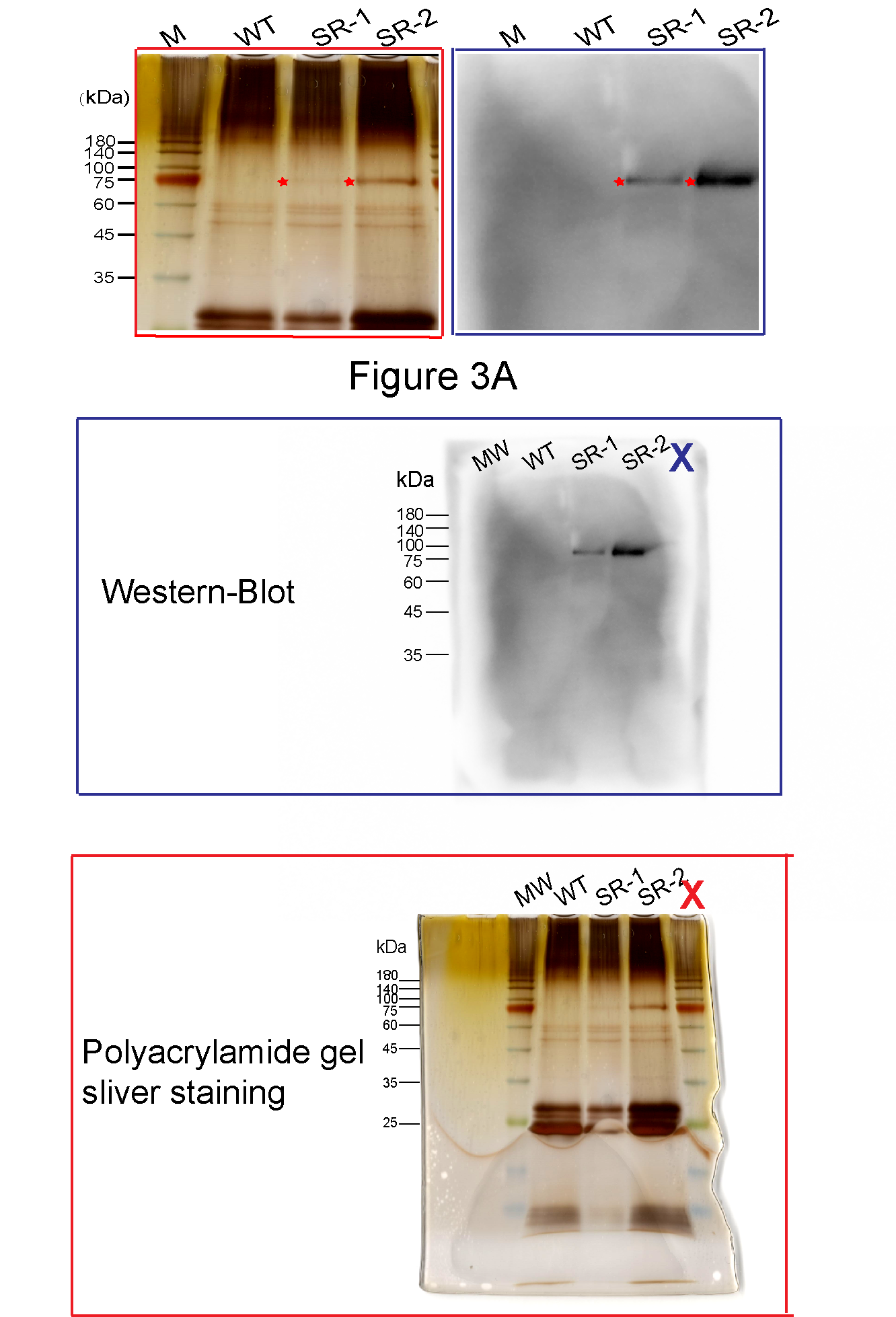

Supplement: S1 Raw images — (TIF) [file pone.0282533.s002.tif]
